# Digital Gene Expression Analysis of Ponkan Mandarin (*Citrus reticulata* Blanco) in Response to Asia Citrus Psyllid-Vectored Huanglongbing Infection

**DOI:** 10.3390/ijms17071063

**Published:** 2016-07-02

**Authors:** Yun Zhong, Chunzhen Cheng, Bo Jiang, Nonghui Jiang, Yongyan Zhang, Minlun Hu, Guangyan Zhong

**Affiliations:** 1 Institute of Fruit Tree Research, Guangdong Academy of Agricultural Sciences, Guangzhou 510640, China; zhongyun99cn@163.com (Y.Z.); ld0532cheng@126.com (C.C.); jb-clinnic@webmail.hzau.edu.cn (B.J.); jiangnonghui2002@163.com (N.J.); fanshu2005@163.com (M.H.); 2 Institute of Horticultural Biotechnology, Fujian Agriculture and Forestry University, Fuzhou 350002, China; zhyy0425@126.com; 3 Key Laboratory of South Subtropical Fruit Biology and Genetic Resource Utilization Ministry of Agriculture, Guangzhou 510640, China

**Keywords:** Asian citrus psyllids (ACP), Citrus Huanglongbing (HLB), ponkan mandarin, differentially expressed genes (DEGs), digital gene expression (DGE)

## Abstract

Citrus Huanglongbing (HLB), the most destructive citrus disease, can be transmitted by psyllids and diseased budwoods. Although the final symptoms of the two main HLB transmission ways were similar and hard to distinguish, the host responses might be different. In this study, the global gene changes in leaves of ponkan (*Citrus reticulata*) mandarin trees following psyllid-transmission of HLB were analyzed at the early symptomatic stage (13 weeks post inoculation, wpi) and late symptomatic stage (26 wpi) using digital gene expression (DGE) profiling. At 13 wpi, 2452 genes were downregulated while only 604 genes were upregulated in HLB infected ponkan leaves but no pathway enrichment was identified. Gene function analysis showed impairment in defense at the early stage of infection. At late stage of 26 wpi, however, differentially expressed genes (DEGs) involved in carbohydrate metabolism, plant defense, hormone signaling, secondary metabolism, transcription regulation were overwhelmingly upregulated, indicating that the defense reactions were eventually activated. The results indicated that HLB bacterial infection significantly influenced ponkan gene expression, and a delayed response of the host to the fast growing bacteria might be responsible for its failure in fighting against the bacteria.

## 1. Introduction

Citrus Huanglongbing (HLB), the most destructive citrus disease, can infect all citrus species, cultivars and hybrids, as well as some citrus relatives. Although the disease has been reported for about 100 years, neither HLB resistant rootstocks and citrus germplasm resources nor effective and durable HLB control methods have so far been explored except the common strategies of removing infected trees, using disease-free nursery trees, and applying an aggressive control program in the management of the vector insect [1,2,3].

The HLB disease is associated with a phloem-limited *α-proteobacterium* designated as *Candidatus* Liberibacter (*Ca*). So far, three forms of *Ca.* Liberibacters, i.e., the Asian form *Ca.* L. asiaticus (*C*Las), the American form *Ca.* L. americanus (*C*Lam) and the African form *Ca.* L. africanus (*C*Laf), have been identified [2], and the *C*Las is the most widespread and severe one. *C*Las and *C*Lam are transmitted by the Asian citrus psyllid (ACP), *Diaphorina* *citri* Kuwayama, while the *C*Laf is transmitted by the African citrus psyllid, *Trioza* *erytrea* Del Guercio. HLB bacteria could also be transmitted by dodders or by grafting with infected budwoods [4]. Once the plant is colonized, HLB bacteria are restricted to the phloem of the host. The phloem is the main traffic route for vital metabolites including carbohydrates, defensive compounds and signaling molecules. Interruption in the transport of these molecules becomes inevitable as the bacteria spread [5]. Nutrient deficiencies and carbohydrate metabolism changes were reported by several studies [6,7]. Nutrient deficiency is found to be one of the typical leaf symptoms in addition to yellowing and asymmetric blotchy mottling [2,5]. Many anatomical disorders such as starch accumulation, callose deposition, phloem plugging and collapse, swelling of sieve element and companion cell walls, and disruption of chloroplast inner grana structures have been observed in HLB infected leaves [2,7,8,9,10,11].

Disease symptom development is considered to be the consequence of molecular, cellular, and physiological changes in the interaction of the host plant with the invading pathogen [12], and understanding the host responses to pathogen infection at a molecular level is therefore very necessary for the clarification of the plant–microbe interaction mechanisms and for the development of novel disease control strategies [13]. In this regard, several studies have been performed on the global gene expression changes in citrus leaves [5,8,13,14,15,16,17], fruits [18,19], stems and roots [20,21], and some gene co-expression network analyses were also conducted to identify candidate citrus genes with potentials of against HLB [22,23,24]. Though a substantial amount of knowledge has been generated, it should be noted that all the HLB-infected citrus trees used in the above studies were from grafting with HLB infected buds or from symptomatic trees in open field. However, the possibility cannot be ruled out that citrus might respond differently to psyllid transmitted and graft-transmitted HLB infections as bacteria titters could differ by more than 1000-fold [25] and times required for a successful transmission also differed a lot between the two transmission pathways [26]. It is, therefore, worth investigating ACP-vectored infection to better understand the HLB pathogenesis.

Ponkan is widely cultivated in Asian countries for its high quality fruits, but ponkan production has been problematic in HLB endemic areas since ponkan trees are highly susceptible to HLB infection. Knowledge about why ponkan trees collapse rapidly following HLB infection is also of great value to the understanding of HLB progression.

In the present study, a digital gene expression (DGE) profiling [27] was used to detect the changes in global gene expression in ponkan mandarin (*C. reticulata* Blanco) leaves following exposing the trees to ACP adults raised on HLB symptomatic trees. The time-course transcriptional changes at the early symptomatic (13 weeks post inoculation, wpi) and symptomatic (26 wpi) stages of *C*Las infection were compared and discussed.

## 2. Results

### 2.1. Development of Citrus Huanglongbing (HLB) Symptoms on Experimental Trees

Three of the five ponkan trees exposed to *C*Las-carrying psyllids were detected to be HLB positive by conventional PCR since 3 wpi while all the controls were HLB negative during the whole experiments (Figure S1). At 13 wpi, blotchy mottling symptom began to appear on some leaves of trees, and at 26 wpi, some HLB positive plants displayed typical symptoms of asymmetrical blotchy mottling. Leaf samples for DGE analysis were then taken from both the *C*Las treated and the control plants at the two time points.

### 2.2. Digital Gene Expression (DGE) Profiling Result

Between 3,587,092 and 3,704,176 raw reads were generated from the two time points of both treatments (Table 1). Approximately 69.59% to 78.09% of the total reads could be successfully mapped to *Citrus clementina* genome, and 54.30% and 64.69% of the distinct tags were mapped to the archived gene sequences (Table 1).

### 2.3. Functional Analysis of DGE Profiling Data

**Figure 1:**
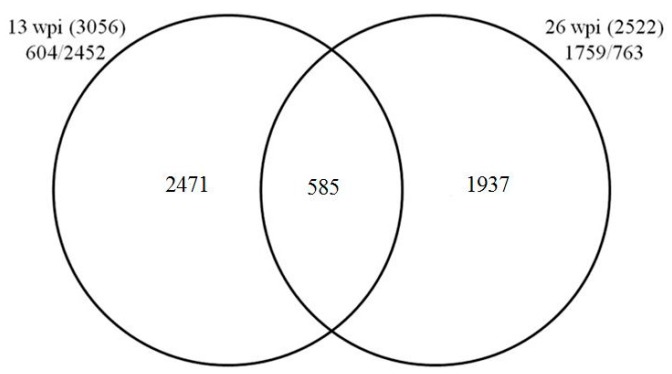
Differential regulation of genes in leaves at 13 and 26 weeks post inoculation (wpi). Figures in the two parentheses indicate total numbers of differentially expressed genes (DEGs) identified in leaves 13 and 26 wpi, respectively. Figures before virgules indicate numbers of upregulated DEGs, and those after virgules indicate the downregulated.

In total, 3056 and 2522 DEGs with fold change ≥1.5 (*p*-value ≤ 0.005 and FDR (false discovery rate) ≤ 0.001) were identified to be regulated at 13 and 26 wpi, respectively. For the 3056 DEGs identified from samples at 13 wpi, 604 were upregulated whereas 2452 were downregulated (Table S1). For the 2522 DEGs identified from samples at 26 wpi (Table S2), 1759 were upregulated while 763 were downregulated. Only 585 DEGs were commonly identified from both time points (Figure 1 and Table S3).

### 2.4. Gene Pathway Enrichment Analysis of HLB-Modulated Pathways

**Figure 2:**
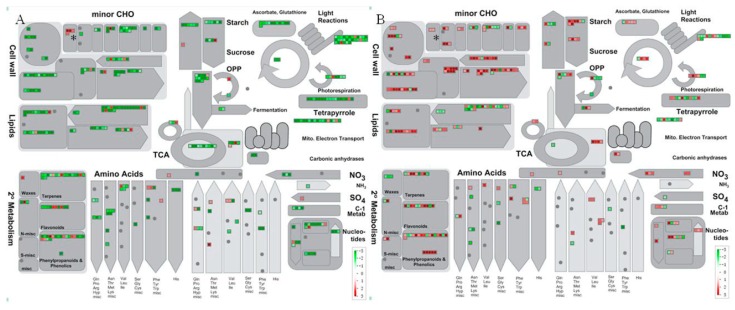
Overview of the DEGs involved in metabolisms at 13 (**A**) and 26 wpi (**B**) respectively in ponkan leaves infected with Asian citrus psyllid (ACP)-vectored *C*Las bacteria. The red show the genes that were significantly upregulated while the green show the significantly downregulated. CHO: Carbohydrate; TCA: Tricarboxylic acid cycle; OPP: Oxidative Pentose Phosphate; * Raffinose family metabolism; Grey dots: genes without significant expression difference, arrows were used to indicate the direction of the pathways.

**Figure 3:**
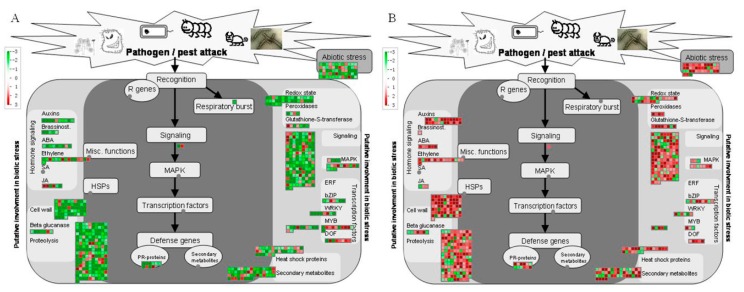
Stress related DEGs regulated by *C*Las infection at 13 wpi (**A**) and 26 wpi (**B**) respectively in ponkan leaves. Genes significantly upregulated by *C*Las infection are displayed in red, and those downregulated are shown in green. HSPs: heat-shock proteins; bZIP: basic leucin zipper; ERF: Ethylene response factor; MAPK: mitogen-activated protein kinase; MYB: MYB family transcription factor; DOF: DNA binding with one finger. ABA: abscisic acid; SA: salicylic acid; JA: jasmonic acid; R gene: resistance gene; Misc.: miscellaneous, PR-protein: pathogenesis-related protein, WRKY: WRKY transcription factors, DOF: DNA-binding with one finger.

PageMan analysis showed that no significantly changed pathway was identified at *p*-value ≤0.05, although more DEGs were found at 13 wpi. The HLB significantly influenced 12 pathways at 26 wpi included protein synthesis, cell wall metabolism, transport, DNA synthesis, secondary metabolism, and auxin involved hormone signaling pathways etc. (Table 2). MapMan gene ontology results showed that these DEGs were mainly involved in diverse cellular functions including carbohydrate metabolism, stress-response-pathways, transport, cell organization, etc. (Figure 2 and Figure 3).

### 2.5. Carbohydrate Metabolism Was Significantly Regulated by HLB Infection

DEGs involved in carbohydrate metabolism were mostly downregulated at early infection stage (13 wpi) but mostly upregulated later at 26 wpi (Figure 2). Two sucrose and starch related DEGs, *sucrose-phosphate synthase* (*SPS*) (ciclev10018655m) and *β-fructofuranosidase/invertase* (ciclev10019134m), were found to be upregulated at both 13 and 26 wpi. Transcripts for 1 AGPase, 2 starch synthase, 1 sucrose-phosphatase 1 (SPP1), and 1 β-amylase were downregulated while other starch degradation related genes were all upregulated at 26 wpi (Table S2).

Notably, four raffinose metabolism related genes including two *galactinol synthase 2*, one *galactinol synthase 4*, and one *galactinol-sucrose galactosyltransferase*, were all found to be significantly induced by HLB infection at 13 wpi. The two *galactinol synthase 2* genes were both upregulated by more than three-fold. Moreover, upregulation of two raffinose biosynthesis related genes, *galactinol synthase 4* (ciclev10032043m) and *stachyose synthase precursor* (ciclev10004372m), were also found at 26 wpi.

At 13 wpi, two *glucan synthases* were downregulated, while three *glucan synthase* genes were upregulated at 26 wpi. Genes involved in trehalose, sugar alcohols, myo-inositol and galactose were all downregulated at 13 wpi but were mostly upregulated at 26 wpi (Tables S1 and S2).

### 2.6. Stressresponse-Related Genes

**Figure 4:**
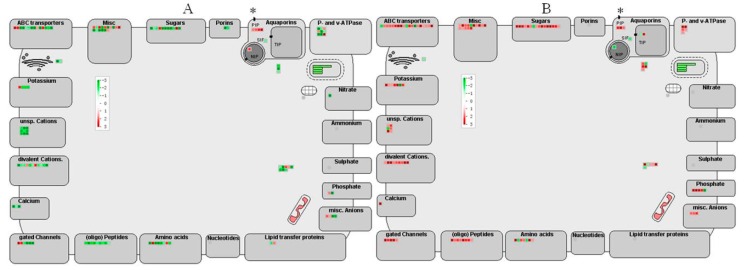
MapMan views of transport related genes that were differentially regulated after *C*Las infection at 13 (**A**) and 26 wpi (**B**). Genes significantly upregulated by *C*Las are displayed in red, and the downregulated are shown in green. * *PIP* (plasma membrane intrinsic protein) genes; *SIP*: small and basic intrinsic protein genes; *NIP*: Nod26-like intrinsic protein genes; *TIP*: tonoplast intrinsic protein genes; grid framed with ellipse represents chloroplast.

MapMan categorized 714 (23.36%) and 622 (24.66%) DEGs into the “stress-related” at 13 and 26 wpi (Figure 4), respectively. Stress response-related pathways such as cell wall, proteolysis, secondary metabolites, signaling and transcription factors, hormone signaling and heat shock protein involved pathways, displayed differential responses in HLB-infected ponkan leaves.

Almost all the cell wall-related genes were initially downregulated at 13 wpi but upregulated later at 26 wpi. At 13 wpi, only 2 of the 10 *pathogenesis-related* (*PR*) *proteins* were upregulated. At 26 wpi, however, 10 of the 12 *PR* DEGs were upregulated, and notably two disease resistance protein genes (ciclev10024511m and ciclev10022439m) were found to be upregulated by 9.5-fold and 11.5-fold, respectively (Tables S1 and S2).

At 13 wpi, 10 of the 23 *MYB transcription factor* DEGs were upregulated. *LHY* (*late elongated hypocotyl*, ciclev10018966m) and *EPR1* (*early-phytochrome responsive 1*, ciclev10020135m) were found to be upregulated by more than four-fold at 13 wpi (Table S1). At 26 wpi, more upregulated transcription factor genes were found in HLB infected samples. However, the same *LHY* gene (ciclev10018966m) was downregulated about two-fold (Table S2).

The expression of secondary metabolism related genes involved in the metabolisms of flavonoids, phenylpropanoids and lignin, isoprenoids and alkaloids was mostly downregulated at 13 wpi but mostly upregulated at 26 wpi (Figure 2). A wax metabolism related gene, *WAX2/CER3* (ciclev10007738m), was upregulated more than two-fold at 13 wpi. However, three wax-related genes including two *CER1* and one *wax synthase-related* were downregulated at 26 wpi. A *GGPS1* (*geranylgeranyl pyrophosphate synthase 1*) was upregulated about 10-fold at 26 wpi. Two *VTE2/HPT1* (*homogentisate* *phytyltransferase 1*) genes were found to be downregulated at 13 wpi. While at 26 wpi, a *VTE2* (ciclev10008336m) was upregulated about eight-fold. The simple phenols metabolism pathway was found to be significantly altered by HLB infection at 26 wpi (Table 2). Two *IRX12* (*irregular xylem 12*) (ciclev10031134m and ciclev10011400m), 1 *LAC11* (*laccase 11*) and 2 *LAC17* (*laccase 17*) genes were significantly induced. The two *IRX12* genes were both highly upregulated about 10-fold at 26 wpi, but one of them (ciclev10031134m) was downregulated more than eight-fold at 13 wpi (Table S3).

Significant transcriptional changes in response to *C*Las infection were observed for a group of genes involved in hormone biosynthesis and signaling (Figure 3). At 13 wpi, most of the hormone-related genes were repressed by HLB infection. However, a gene of 2-oxoglutarate (*2OG*) and Fe(II)-independent oxygenase (*2OG-Fe(II) oxygenase*) was found to be upregulated by more than 11-fold (Table S1). Moreover, three jasmonic acid(JA) synthesis related *lipoxygenase* genes were upregulated at 13 wpi. Auxin metabolism was found to be significantly modulated by HLB infection at 26 wpi with most of the involved genes upregulated. Ethylene and JA related DEGs were mostly upregulated at 26 wpi.

Signaling related DEGs encoding receptor like kinases (RLK), calcium modulating proteins and G-proteins were mainly downregulated at 13 wpi but upregulated at 26 wpi (Table S3). However, 3 *RLK* genes were upregulated by more than 8-fold at 13 wpi.

Protein synthesis related genes were overwhelmingly repressed by HLB infection at both 13 and 26 wpi (Table S3). The expression of many *heat shock proteins* was suppressed at 13 wpi, while several *HSP70* genes were found to be upregulated at both 13 and 26 wpi.

### 2.7. Transport Related Genes Regulated by HLB Infection

One hundred and twenty eight and 126 transport related DEGs were identified in HLB infected ponkan at 13 and 26 wpi, respectively (Figure 4, Tables S1 and S2). Most of the DEGs were downregulated at 13 wpi but induced at 26 wpi, but 4 *PIP*s (*plasma membrane intrinsic protein*) were upregulated at 13 wpi.

### 2.8. Cell Organization Related Genes

Many cell organization related genes were also found to be regulated by HLB infection and most of them were downregulated at 13 wpi but upregulated at 26 wpi (Table S3). Notably, two *phloem protein* genes (*PP*), *PP2-B2* (ciclev10005659m) and *PP2A-1* (ciclev10016359m), were upregulated at 13 wpi and another *PP* gene, *PP2-A12*, was upregulated at 26 wpi.

### 2.9. Quantitative Real Time PCR (qRT-PCR) Result

**Figure 5:**
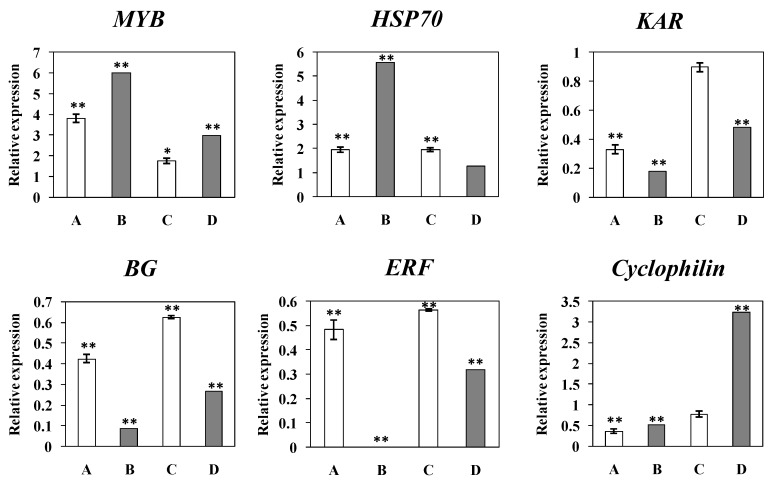
Comparison of the expression levels in ponkan leaves infected with *C*Las relative to their controls of six of the representative genes obtained by real-time PCR (A,C) and by digital gene expression (DGE) profiles (B,D). A,B: at 13 wpi; C,D: at 26 wpi. * and ** indicate significant changes at *p* < 0.05 and *p* < 0.01, respectively. *MYB*, MYB family transcription factor; HSP, heat-shock proteins; *KAR*, ketol-acid reductoisomerase; *BG*, β-1,3-glucanase; *ERF*, Ethylene response factor.

To validate the DGE data, quantitative Real Time PCR (qRT-PCR) was performed to investigate the transcriptional patterns of six representative genes as shown in Figure 5. The upregulated expression patterns of the *MYB* and *HSP70* genes detected by qRT-PCR were in accordance with the DEG profile results. Similarly, the qRT-PCR detected downregulation in the expression of *ERF*, *KAR*, *β-1,3-glucanase* was also in accordance with the DGE profile results that showed the three genes were downregulated more significantly at 13 wpi than at 26 wpi. *Cyclophilin* was also found to be downregulated at 13 wpi by both qRT-PCR and DGE analysis, although it showed still a downregulation by qRT-PCR but an upregulation by DEG data at 26 wpi. Moreover, the temporal expression trends for all qRT-PCR verified genes were generally in agreement with those shown by DEG profiling data.

## 3. Discussion

Most of the previous reports on the early responses of citrus to *C*Las infection used graft-transmitted citrus materials [11,14,15,16,17,20,21]. Obviously, graft-transmission has been largely responsible for the wide and rapid spread of the disease in many countries [5]. However, the disease is naturally transmitted by ACP in open fields though it can be vectored by dodders in neglected orchards. Numerous reports have shown that inoculation by grafting with HLB-carrying budwoods required 50 days [21] to more than two months [26] for the bacteria to grow to a detectable level. But it is indicated that ACP was a more efficient vector, for HLB bacteria could be detected in less than seven days in leaves of jasmine oranges after exposing the trees to HLB-carrying ACPs [26]. In this study, we also found that psyllids were an efficient vector since HLB bacteria were detected within less than 3 wpi by conventional PCR.

Given the importance of leaves in photosynthesis and export of carbohydrates and in HLB symptom development, changes in gene expression in HLB infected citrus was first investigated in *C*Las infected Valencia orange (*Citrus sinensis* L. Osbeck) leaves in 2008 by Albrecht and Bowman [14]. Later, many studies were also performed by using *C*Las infected leaves of sweet oranges (*C. sinensis*), Cleopatra mandarins (*C.* *reticulata*), and rough lemon (*C. jambhiri*) in various disease development stages [8,15,16,17]. In this study, we extended the research to ponkan, a very important mandarin cultivar that had dominated the citrus production in Guangdong province of China in the 1980s and was almost totally wiped out by HLB in late 1990s. With the use of DGE profiling we were able to find the dramatic differences between the transcriptomes of leaves from HLB infected and healthy ponkan trees at two representative time points, 13 and 26 wpi.

Previous studies reported that HLB-infection evoked defensive reactions in citrus for defense genes were general mostly upregulated [11,15,21]. We also found that ACP-transmitted HLB infection induced an overwhelmingly defensive reaction in ponkan leaves at 26 wpi as shown by the upregulation in many genes involved in cell-wall modifications, protein synthesis, transport, DNA synthesis, secondary metabolism, and auxin involved hormone signaling pathways (Table 2). However, a different picture was shown as we examine our data obtained from 13 wpi, which clearly showed that a substantial number of ponkan defense genes responded to *C*Las by downregulating their expression at this early stage. The significance of the finding is that ponkan’s defense waned at the early stage, possibly from a rapid build up in *C*Las population in consideration of a more natural vector, ACP, was used in our study.

Carbohydrates account for a large part of the translocated materials in phloem, and sucrose was reported to be the predominant sugar in phloem sieve tubes [28]. In our study, *SPS* gene (ciclev10018655m) and *β-fructofuranosidase*/*invertase* gene (ciclev10019134m) were the two sucrose and starch metabolism related genes that were upregulated at both 13 and 26 wpi. Invertase was reported to play a key role in the activation of stress responses and may function as an extracellular indicator for pathogen infection [29,30]. AGPase (ADP-glucose pyrophosphorylase) is the key enzyme catalyzing the rate-limiting step in starch biosynthesis [31], and the repression of *AGPase* and the upregulation of starch degradation related genes at 26 wpi may be a feedback regulation from accumulated starches in leaves. Other sugar metabolism pathways were also found to be affected by HLB infection. Galactinol and raffinose can protect plants from oxidative damage [32]. Galactinol synthase is the first enzyme in raffinose synthesis and regulates the partitioning between sucrose and raffinose, and was hypothesized to function in reducing sucrose level in phloem of HLB infected leaves and in scavenging reactive oxygen species (ROS) [5]. The significant upregulation of the two galactinol synthase genes suggested that the sucrose levels might be accumulated following HLB-infection, possibly from the above mentioned increase in invertase activity.

*C*Las infection has been suggested to result in blockage of translocation [10]. The blockage comes partly from deposition of callose (β-1,3 glucan), which has been repeatedly observed in the sieve pores [7,33]. Callose synthesis can be induced by diverse biotic and abiotic stresses [33]. It has also been shown that mutant plants in which phloem transport is inhibited accumulate excessive callose around plasmodesmata in their phloem cell walls [34]. In *C*Las infected citrus leaves, excessive callose formation in the phloem plasmodesmata preceded starch accumulation [33]. The upregulation of glucan synthase genes at a late stage might be related to callose deposition in HLB infected ponkan leaves. In addition, accumulation of phloem specific proteins at the sieve plates is also generally considered to cause the blockage of translocation stream [35]. In this respect, we found two upregulated phloem protein genes (*PP*), *PP2-B2* and *PP2A-1* at 13 wpi and another upregulated *PP* gene, *PP2-A12*, at 26 wpi, and similar findings were reported in other studies [8,14,15].

Cell walls constitute the first defense barrier, protecting plants from bacterial infection. Cell wall metabolism was identified to be one of the most dramatically altered pathways in HLB-infected citrus trees [5,8,13,14,15,16,17,18,19,20,21,22]. In this study, the cell wall-related genes were mostly downregulated at 13 wpi. Among them are *IRX* genes that are involved in xylan biosynthesis and were reported to function in building the xylan backbone in the secondary and primary cell walls [36,37]. At 13 wpi, a *IRX 12* was downregulated by more than eight-fold. At 26 wpi, however, two *IRX12* were upregulated about 10-fold. In addition, many other cell-wall related genes were induced at 26 wpi including two significantly induced laccase genes. It is proposed that laccases play a role in the formation of lignin by promoting the oxidative coupling of monolignols [38].

PR proteins play important roles in plant defense against pathogens. At 13 wpi, most PR protein genes were found to be downregulated. It was reported that suppression of host defenses was critical for pathogenesis [39], and hence it is conceivable that the suppression of *PR* genes should favor *C*Las infection. At 26 wpi, however, most of the *PR* genes were upregulated, which might be an indication for the eventual activation of defense mechanisms that lead to processes such as callose deposition in and around phloem tissues [40].

Some transcription factors, such as WRKYs and MYBs, could bind to promoter elements of individual defense related genes and were closely related to plant defense [8]. MYB transcription factors play important roles in many processes including stress response [41]. At 13 wpi, a *LHY* and an *EPR1* were upregulated by more than four-fold. These two genes were reported to play critical roles in regulating circadian rhythm [42,43]. Altered expression of these genes can cause abnormal circadian rhythms which, in turn, would lead to the disorders of clock-regulated pathways, such as photosynthesis, transport of sugars, and starch metabolism [17,44]. Correspondingly, the repression in photosynthesis and carbohydrate metabolism has been observed in HLB infected ponkan at 13 wpi. At 26 wpi, however, the same *LHY* gene (ciclev10018966m) was downregulated about two-fold, which may indicate an eventual failure in the regulation of these vital processes.

The expression of secondary metabolism related genes were mostly downregulated at 13 wpi but mostly upregulated at 26 wpi. However, one wax metabolism related gene *WAX2/CER3* (ciclev10007738m) was upregulated more than two-fold while two *cerberus1* (*CER1*) were downregulated at 26 wpi. The *Arabidopsiswax2* is involved in cuticle membrane and wax production [45,46]. *CER1* expression was reported to be induced by osmotic stresses and regulated by abscisic acid [47]. However, *CER1* over-expression could increase susceptibility to bacterial and fungal pathogens [47]. The downregulation of *CER1* at 26 wpi might be important for increasing the tolerance of ponkan to *C*Las infection.

Vitamin E deficient 2 (VTE2) is the first enzyme of tocopherol biosynthesis pathways and is involved in phloem sucrose loading [48]. The loss of VTE2 function-mutants leaded to a phenotype that resembles HLB symptom in citrus [5]. *VTE2* was downregulated in asymptomatic *C*Lam-infected citrus leaves [5] as shown in our study at 13 wpi, but significantly upregulated at 26 wpi. The downregulation of *VTE2* in the early stage (13 wpi) might contribute to HLB symptom development and the upregulation of it in the late stage (26 wpi) might indicate a need for restoring normal phloem translocation of nutrients.

Hormone biosynthesis and signaling were significantly regulated by HLB infection. 2OG-Fe(II) oxygenase is involved in biosynthesis of secondary metabolites including flavonoids and gibberellins. The basal expression of this gene was also reported to be much higher in HLB tolerant species US-897 [15]. The significant upregulation of *2OG-Fe(II) oxygenase* at 13 wpi might play a role in resistance against HLB. Auxins play a key role in plant development and the auxin response pathway is connected to the SA, JA, and ethylene (ET) signaling network in various ways [13]. Auxin metabolism was found to be significantly modulated by HLB infection at 26 wpi. At the same time, most of the ethylene and JA related DEGs were found to be upregulated. *GGPS1*, catalyzing the formation of geranylgeranyl pyrophosphate, was reported to be induced by JA- or methyl salicylate (MeSA)- treatment [49], and a transcript of *GGPS1* was upregulated about 10-fold at 26 wpi. Put together, a complex hormone regulation should be involved in the response of ponkan to HLB infection.

RLKs, calcium modulating proteins and G-proteins were involved in signaling and regulation of plant defense and defense response, and the expression of these genes was not only significantly regulated in HLB infected ponkan leaves as shown in our study but was also identified in HLB infected fruits and roots [1,21].

The ubiquitin/proteasome system (UPS) has been involved in the signaling transduction of stimuli and in the perception and signaling of plant hormones [50]. Several *HSP70* genes, which are involved in signal transduction leading to plant defense responses [51], were found to be upregulated at both 13 and 26 wpi. We also found that the expression of many other heat shock proteins was suppressed at 13 wpi, and a reduction in HSP proteins was thought to be responsible for increased protein misfolding [19].

### Transport Related Genes Regulated by HLB Infection

Four *PIPs* genes were found to be upregulated at 13 wpi. PIPs are aquaporins and are responsible for passive transmembrane transport of water and other small molecules [52]. Yeast cells expressing *Vitis* aquaporins were reported to be osmosensitive [53]. Swelling in the middle lamella was observed in the presymptomatic leaves of *C*Las infected trees [7]. The upregulation of these genes might function in promoting water and mineral transport in HLB infected ponkan leaves at the early stage of infection. The upregulation of transport related DEGs at 26 wpi might be driven by nutrient deficiency, which contrasts their counterparts that showed repression in *C*Las infected sweet orange shoots [20].

In conclusion, our result showed that ACP-transmitted *C*Las infection weakened quickly the defense of ponkan as shown by reduced expression of many defense genes at early stage (13 wpi), and a seeming reactivation of defense at symptomatic stage of 26 wpi since DEG data obtained at 26 wpi showed an upregulation in most genes involved in carbohydrate metabolism, plant defense, hormone signaling, secondary metabolism, transcription regulation, and other pathways.

## 4. Materials and Methods

### 4.1. Asian Citrus Psyllids Collection and Feeding

ACP adults were collected from new flushes of jasmine orange (*Murraya* *paniculata*) in spring of 2009 and used to establish a laboratory colony. The colony was maintained on disease-free jasmine orange seedlings in net cages. Eggs were transferred to disease-free jasmine orange seedlings to get disease-free ACPs, and the fourth instar nymphs were transferred to *C*Las-positive Hongjiangcheng (*Citrus sinensis* Osbeck cv. “Hongjiangcheng”) trees for 20 days to obtain *C*Las-carrying ACP adults. All the experiments were conducted under screen houses.

### 4.2. HLB Inoculation and Detection

Ponkan seeds were sown in sterilized soil in February 2008, and were used for the experiment at 31 August 2009. Five ponkan seedlings were exposed to 40 *C*Las-carrying ACP adults for 3 days. For controls, 5 ponkan seedlings were exposed to 40 disease-free ACP adults. PCR was performed every week to detect the presence of *C*Las in leaves of the samples using A2/J5 primers [54].

### 4.3. RNA Preparation and Digital Gene Expression Profiling

Fully expanded leaves were sampled from three HLB positive trees, respectively, at 13 wpi when chlorosis was barely discernible at the lower leaf areas, and at 26 wpi when typical mottling chlorosis symptoms developed. Leaf samples were also collected from the control trees. Total RNA was extracted using Trizol reagent according to manufacturer's instruction and treated with RNase-free DNase I (TaKaRa, Dalian, China) to remove contaminated DNA. The quality and quantity of the purified RNA were determined by measuring the absorbance at 260/280 nm (A260/A280) using NanoDrop 2000c UV-Vis Spectrophotometer (Thermo Scientific, Wilmington, DE, USA). RNA integrity was as assessed on 1% agarose gels. RNA samples were then pooled by treatment by taking an equal amount of RNA aliquot from each of the three RNA samples of the same treatment, and the four pooled samples were sent to the Beijing Genomics Institute (BGI; Shenzhen, China) for digital gene expression profiling. Sequencing was carried on Illumina HiSeq™ 2000 platform by using sequencing by synthesis (SBS) method. Millions of 35 nt (CATG + 17 nt gene sequence + 14 nt 3′ adaptor sequence) long raw sequences were generated.

### 4.4. Differential Gene Expression Analysis

Clean reads were obtained from raw sequencing data by removing the reads containing adapter or ploy-N and those of low quality. These reads were aligned to *Citrus clementina* gene sequences (available at: http://www.phytozome.org.) using SOAP2 [55] in 2011. Number of distinct gene sequences was calculated using total numbers of clean tags that could be perfectly mapped. TPM (Transcripts Per Million) was used for relative assessment of gene expression levels. DEGs were identified by the DESeq R package [56]. Corrected *p*-value < 0.005, FDR ≤ 0.001 and the absolute value of log_2_(ratio) ≥ 1.5 were set as the thresholds for identifying significantly differential expressed genes. Two pairwise comparisons were made between samples of the HLB infected and the control samples collected at 13 wpi and at 26 wpi.

All the DEGs with gene IDs of the old version (Cclementina_165) were once again aligned with the new version of Clementine genome (Cclementina_182) and used for MapMan analysis in 2014. Pathway analysis was performed using PageMan embedded in the free-downloadable software MapMan [57]. A Wilcoxon test was applied and a statistics-based overview of changed pathways from global gene expression alteration was provided. The MapMan program was used to map the DEGs into specific pathways.

The raw data have been submitted to NCBI (BioProject ID: PRJNA318724).

### 4.5. Quantitative Real Time PCR (qRT-PCR) Analysis

To validate results obtained by digital gene expression profiles, aliquots of the same RNA samples were subjected to qRT-PCR analysis. Six representative genes including MYB transcription factor (MYB, ciclev10012263m), heat shock protein 70 (HSP70, ciclev10030928m), ERF transcription factor (ERF, ciclev10021285m), Ketol-acid reductoisomerase (KAR, ciclev10014720m), β-1,3-glucanase (B1,3G, ciclev10008176m) and cyclophilin (ciclev10029386m) were analyzed. cDNA was reverse-transcribed from the RNA samples using All-in-One™ qPCR Mix (GeneCopoeia) PrimeScript RT reagent kit (TaKaRa, Dalian, China). Primers specific to the 6 genes were designed with the Primer3 [58] and synthesized by BGI. Primers of internal reference gene β-actin were designed according to Cheng et al. [59]. All the primer sequences were listed in Table S4. The RT-qPCR was carried out on the thermocyclerCFX96 (Bio-Rad, Hercules, CA, USA) in a final volume of 20 µL containing 2 µL of cDNA, 10 µL 2× All-in-One™ (Genecopoeia, Rockville, MD, USA), 1 µL each of the forward and the reverse primers (10 µM), and 6 µL of sterile water. The thermocycler was programmed as: 95 °C for 10 min followed by 40 cycles of 95 °C for 5 s, 60 °C for 10 s and 72 °C for 15 s. The expression was calculated by 2^−ΔΔ*C*t^ method [60] and normalized with the results of β-actin gene [59]. The amplification efficiency and the sizes of the amplicons were listed in Table S4. The curves of the first derivatives of the original qRT-PCR amplicons were single peaks (data not shown), indicating the quality of the PCR was acceptable. The results were shown as mean ± standard deviation from triplicate experiments. The significance of gene expression difference was calculated using *t*-test based on the Δ*C*_t_ values [61], which was carried out by SPSS version 19 (IBM, Chicago, IL, USA).

## 5. Conclusions

Global gene expression profiling reveled that there were more down-regulated than up-regulated genes in leaves of ponkan (*C. reticulata*) mandarin trees following psyllid-transmission of HLB at 13 wpi, and classification of the affected genes indicated that the defense of the trees at the early stage of infection was compromised. However, the defensive responses were apparently activated at later stage of 26 wpi as was shown by changes in the expression of defense-related genes. The results indicated that a delayed defensive response to the fast growing bacteria might be responsible for the failure of Ponkan in fighting against HLB infection.

## Figures and Tables

**Table 1:** Statistical results of digital gene expression profiles. M1 and M2: control ponkan leaves sampled at 13 and 26 weeks post inoculation (wpi), respectively; H1 and H2: Citrus Huanglongbing infected ponkan leaves sampled at 13 and 26 wpi, respectively.

Summary Detail	M1	M2	H1	H2
Number of total raw tags	3,587,092	3,696,000	3,704,176	3,587,500
Number of distinct tags	200,301	207,328	191,280	230,525
Number of total clean tags	3,479,412	3,558,497	3,602,015	3,449,820
Number of distinct tags	92,796	89,297	89,324	110,577
Total clean tags mapped to Gene	2,715,908	2,778,658	2,506,722	2,668,438
Percentage of clean tags mapped to Gene	78.06%	78.09%	69.59%	77.35%
Number of distinct tags mapped to Gene	55,259	57,768	48,500	68,912
Percentage of distinct tags mapped to Gene	59.55%	64.69%	54.30%	62.32%

**Table 2:** HLB-modulated pathways in ponkan leaves, showing the 12 pathways significantly changed following HLB infection at 26 wpi (*p*-value ≤ 0.05).

Bin ID	Pathway Name	DEG Numbers	*p*-Value
29.2.1	protein. synthesis. ribosomal protein	40	2.81 × 10^−^^7^
29.2	protein. synthesis	55	1.22 × 10^−6^
10	cell wall	71	6.35 × 10^−5^
29.2.1.2	protein. synthesis. ribosomal protein. eukaryotic	21	3.46 × 10^−4^
34	transport	126	5.49 × 10^−3^
28.1	DNA. synthesis/chromatin structure	26	7.96 × 10^−3^
29.2.1.2.1	protein. synthesis. ribosomal protein. Eukaryotic. 40S subunit	11	2.24 × 10^−2^
28.1.3	DNA. synthesis/chromatin structure. histone	7	2.65 × 10^−2^
10.2	cell wall. cellulose synthesis	9	3.18 × 10^−2^
16.1	secondary metabolism. simple phenols	5	4.46 × 10^−2^
29.2.1.1	protein. synthesis. ribosomal protein. prokaryotic	15	4.46 × 10^−2^
17.2	hormone metabolism. auxin	24	4.62 × 10^−2^

Bin is the unit used in Mapman graphs denoting a pathway, organelles or gene, and each bin has been assigned a specific ID in Mapman; DEG, differentially expressed gene. Dots in Pathway Name are in accordance with the dots showing in the Bin ID column.

## Supplementary Materials

Supplementary materials can be found at http://www.mdpi.com/1422-0067/17/7/1063/s1. Figure S1: PCR detection for Citrus Huanglongbing (HLB) bacteria in leaves at 3 wpi, Table S1: Mapman analysis result of the 3056 DEGs identified at 13 wpi, Table S2: Mapman analysis result of the 2522 DEGs identified at 26 wpi, Table S3: Mapman analysis result of the 585 DEGs identified at both 13 and 26 wpi, Table S4: Primers used for Real Time PCR analysis.

## Abbreviations

HLB	Citrus Huanglongbing
WPI	weeks post inoculation
DGE	digital gene expression
ACP	Asian citrus psyllids
DEG	Differentially expressed gene
*C*Las	*Candidatus* L. asiaticus
*C*lam	*Candidatus* L. americanus
*C*Laf	*Candidatus* L. africanus
SPS	sucrose-phosphate synthase
SPP1	sucrose-phosphatase 1
LHY	late elongated hypocotyls
EPR1	early-phytochrome responsive 1
GGPS1	geranylgeranyl pyrophosphate synthase 1
HPT1	homogentisate phytyl transferase 1
IRX12	irregular xylem 12
LAC	laccase
RLK	receptor like kinases
HSP	heat shock protein
PIP	plasma membrane intrinsic protein
PP	phloem protein
UPS	ubiquitin/proteasome system
