# Supplementary material for: Digital Gene Expression Analysis of Ponkan Mandarin (Citrus reticulata Blanco) in Response to Asia Citrus Psyllid-Vectored Huanglongbing Infection
Source: Int J Mol Sci. 2016 Jul 2;17(7):1063. doi: 10.3390/ijms17071063 (PMC4964439; doi:10.3390/ijms17071063)
Supplement: Supplementary file 1 [file ijms-17-01063-s001.zip › ijms-123890-Supplementary Materials/ijms-123890-Supplementary Materials.pdf]

Supplementary Materials: Digital Gene Expression Analysis of Ponkan Mandarin (*Citrus reticulata* Blanco) in Response to Asia Citrus Psyllid-Vectored Huanglongbing Infection

Yun Zhong, Chunzhen Cheng, Bo Jiang, Nonghui Jiang, Yongyan Zhang, Minlun Hu and Guangyan Zhong

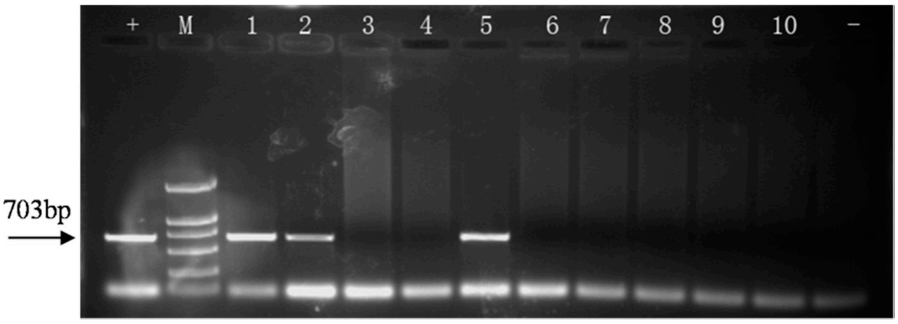

Figure S1. PCR detection for Citrus Huanglongbing (HLB) bacteria in leaves at 3 wpi. +: HLB positive control; −: HLB negative control; 1–5: ponkan seedlings exposed to *C*Las-carrying psyllids; 6–10: ponkan seedlings exposed to *C*Las-free psyllids; M, DNA ladder marker 2000; arrow located the amplification target.
